# Grape Seeds Proanthocyanidins: Advanced Technological Preparation and Analytical Characterization

**DOI:** 10.3390/antiox10030418

**Published:** 2021-03-09

**Authors:** Paolo Morazzoni, Paola Vanzani, Sandro Santinello, Antonina Gucciardi, Lucio Zennaro, Giovanni Miotto, Fulvio Ursini

**Affiliations:** 1 Distillerie Bonollo Umberto S.p.A., Nutraceutical Division, Mestrino, 35035 Padova, Italy; paolo.morazzoni@bonollo.it (P.M.); sandro.santinello@bonollo.it (S.S.); 2 Department of Molecular Medicine, University of Padova, 35129 Padova, Italy; paola.vanzani@unipd.it (P.V.); antonina.gucciardi@unipd.it (A.G.); lucio.zennaro@unipd.it (L.Z.); 3 Proteomics Center, University of Padova and Azienda Ospedaliera di Padova, 35129 Padova, Italy; 4 CRIBI Biotechnology Center, University of Padova, 35129 Padova, Italy

**Keywords:** grapeseed extract, proanthocyanidins, catechins, gel permeation chromatography, ESI-Q-TOF mass spectrometry

## Abstract

A “green” solvent-free industrial process (patent pending) is here described for a grape seed extract (GSE) preparation (Ecovitis™) obtained from selected seeds of Veneto region wineries, in the northeast of Italy, by water and selective tangential flow filtration at different porosity. Since a comprehensive, non-ambiguous characterization of GSE is still a difficult task, we resorted to using an integrated combination of gel permeation chromatography (GPC) and electrospray ionization high resolution mass spectrometry (ESI-HRMS). By calibration of retention time and spectroscopic quantification of catechin as chromophore, we succeeded in quantifying GPC polymers up to traces at *n* = 30. The MS analysis carried out by the ESI-HRMS method by direct-infusion allows the detection of more than 70 species, at different polymerization and galloylation, up to *n* = 13. This sensitivity took advantage of the nanoscale shotgun approach, although paying the limit of missed separation of stereoisomers. GPC and MS approaches were remarkably well cross-validated by overlapping results. This simple integrated analytical approach has been used for quality control of the production of Ecovitis™. The emerging feature of Ecovitis™ vs. a popular benchmark in the market, produced by a different technology, is the much lower content of species at low *n* and the corresponding increase of species at high *n*.

## 1. Introduction

*Vitis vinifera* L. is the most known of the about 900 species present in the Vitaceae family and is also the most important in terms of commercial utilization, mostly based on the use of the fruits and/or their parts (seeds and skins) in the food and medicinal markets.

Grape seed extracts (GSEs) containing variable amounts of polyphenols and so-called oligomeric proanthocyanidins (PACs) have been investigated in several health conditions, including cardiovascular diseases (CVDs) prevention, chronic venous insufficiency (CVI) management, type 2 diabetes. Several clinical studies are now available, depicting their efficacy [1,2,3,4,5,6,7,8,9,10].

Additionally, the leaves, even if for a lesser extent, are traditionally used for medicinal purposes [11,12].

Both fruits- and leaves-derived products are currently mentioned in many Pharmacopeias including the British (1st–3rd Ed.), the US Pharmacopoeias (1st–6th Ed.), and reviewed in agencies monographs such as the European Union herbal monograph edited by the Committee on Herbal Medicinal Product (HMPC) of the European Medicines Agency (EMA).

As previously mentioned, PACs are commonly considered by far the most important group of biologically active polyphenols present in GSEs. They are constituted by a variable number of flavan-3,4-diols (catechin and epicatechin) units with C4-C6 or C4-C8 bonds [9,13]. Typically, PACs present in the seeds, also as gallic acid esters, are of the B-Type, being the simplest dimeric forms represented by PACs B1-B4 and B5-B8. Besides the dimeric forms, PACs are commonly present in the seeds as trimers, tetramers, and larger oligomers.

The presence of B-type dimers, trimers, tetramers, and polymers of up to the size of a dodecamer trigallate was described by Weber et al., who analyzed different commercial GSEs found that the molecular distribution varied substantially [14]. An additional feature of commercially available GSEs is the presence of free or gallic acid-esterified monomeric catechins (catechin and epicatechin) whose content, ranging from 15 to 30%, relays on both maturation stage of grape and extraction procedure. The relevance of monomeric catechins for the clinical efficacy of GSEs is still considered a pending issue. Recent data credibly sustain that monomeric catechins are in a very low percentage rapidly absorbed in the small intestine and then able to reach in a very low concentration the systemic circulation as phase II conjugates. On the other side, oligo-polymeric catechins are extensively metabolized by the colonic gut microbiota into catabolites such as phenyl-γ-valerolactones and their related phenylacetic acids. These intestinal catabolites are described as the most relevant circulating compounds in humans and possibly related to health benefits [15]. This innovative interpretation of oligo-polymeric catechins biological effects has been recently reported for a cranberry extract where valerolactones/valeric acids catabolites have elegantly demonstrated to be responsible for the observed clinical outcomes [16].

Based on these figures, reduced content of monomeric catechins, associated with a higher degree of polymerization, could be seen as a peculiar feature of a GSE be associated with a different, seemingly higher, efficacy when applied in a nutraceutical field. An interesting observation was the effect of grape seed PACs on epithelial cell growth not reproduced by monomeric forms [17].

This paper describes the setting up and the analytical characterization of a new grape seed extract (Ecovitis™) featuring low monomeric catechins content and a high concentration of oligo-polymeric procyanidins. The extract is prepared using selected seeds obtained from Northeast Italian wineries and an extractive food-related procedure based on the sequential combination of an aqueous-infusion and tangential-flow filtration with membranes with varying degrees of selective porosity.

For an in-depth analytical characterization of Ecovitis™ we set up an innovative procedure integrating information from gel permeation chromatography (GPC) and mass spectrometry (MS).

## 2. Materials and Methods

### 2.1. Chemicals and Reagents

BHT (2,6-bis (1,1-dimethylethyl)-4-methylphenol), lithium bromide (LiBr), (+)-catechin (C), (−)-epicatechin (EC), gallic acid (GA), tannic acid (TA), procyanidin B1 (PC-B1), procyanidin B2 (PC-B2), and procyanidin C1 (PC-C1) were bought from Sigma-Aldrich (Milan, Italy); and procyanidin D (PC-D) and procyanidin E (PC-E) from Planta Analytica (New Milford CT, USA). Epigallocatechin (EGC), epicatechin gallate (ECG), and epigallocatechin gallate (EGCG) were from Extrasynthese (Genay, France). All standards purity ranged between 90 and 99%, except for the procyanidin E, which was 87%. InfinityLab EasiVial polystyrene standards calibration kit was purchased from Agilent Technologies (Milan, Italy).

Tetrahydrofuran (THF) HPLC grade and methanol LC/MS grade were purchased from VWR (Milan, Italy). Acetic acid (>99.8%) and phosphoric acid were from Fluka (Milan, Italy). Ultrapure water was prepared from distilled water using a Millipore Milli-Q system (Bedford, MA, USA).

Enovita^®^ has been obtained by Indena S.A.S. (38 avenue Gustave Eiffel, 37095 Tours, Cedex 2, France).

### 2.2. Grape Seeds Extract (Ecovitis*™*) Preparation

**Figure 1:**
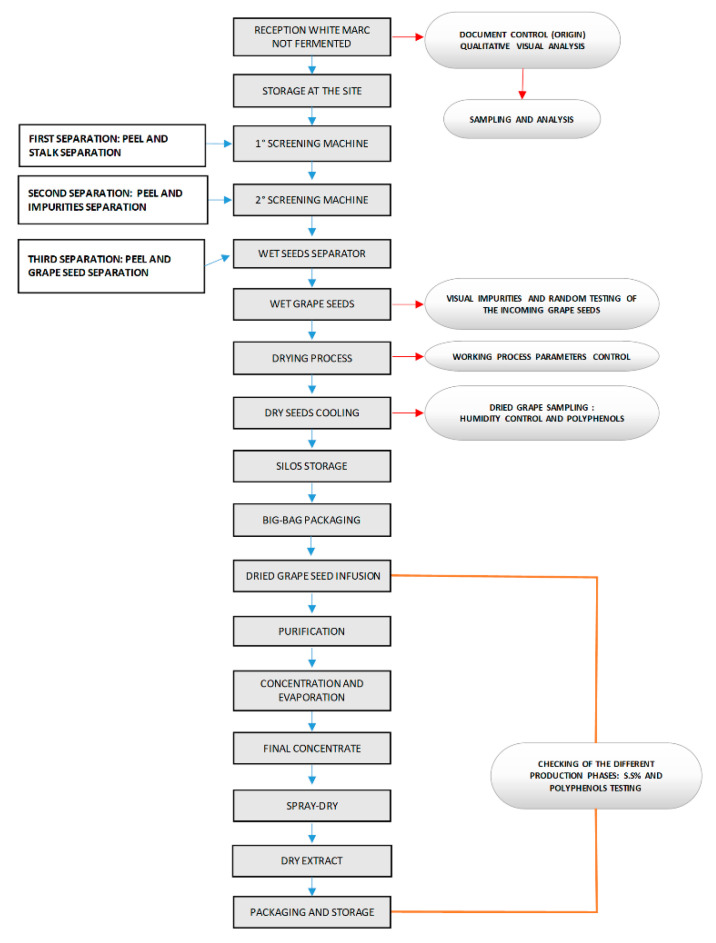
Flow chart of the preparation of grape seed extract (GSE) Ecovitis^TM^.

A brief description of the grape seed extract’s relevant industrial preparative steps (Figure 1) is reported (patent pending).

Selected grape pomaces coming after grape pressing from prescreened Northeast Italian winemakers are immediately transferred to the production plant. Two sequential screening machines are separating seeds from peels and stalks. Seeds are then dried at 70–110 °C and cooled at 30–60 °C with parallel control of residual humidity (less than 8%). Seeds are then analyzed and selected for their content in total procyanidins (≥5%) by the Bates-Smith colorimetric test. Selected dried seeds are now submitted (at room pressure) for 2 h to infusion with hot water (90 °C) in a specific apparatus provided with an internal automatic stirring device activated every 15 min for 1 min. The milieu is controlled continuously for pH and maintained at 3–3.5 employing phosphoric acid. At the end of the infusion (which can also be repeated for an additional cycle), the aqueous phase is separated from the seeds by decantation and further purified by two tangential filtration passages through 0.2-micron membranes (microfiltration). The permeate is then submitted to an elution on specific pyrolyzed absorption resins for the complete elimination of eventual contaminants regarding the relevant existing legislation (e.g., EC N. 396/2005; US CFR Title 40, Part 180). The obtained permeate is then submitted to ultrafiltration on membranes with 300 kDalton cut-off.

The permeate here is discarded, and the retentate rich in PACs is submitted to an additional step of ultrafiltration using hot water (90 °C). The final retentate is then submitted to tangential nanofiltration to decrease water content before under-vacuum concentration at 25 °C. The last step is a spray-drying process conducted using an entrance temperature of 190 °C, an exit temperature of 80 °C, a timing of contact with air of 2 s, and an air volume/retentate weight ratio of 300 m^3^/kg. The obtained dry extract is finally submitted to a double-sealed aluminum packaging. In these conditions, the dry extract has demonstrated good 3-year stability in all the conventional tests.

### 2.3. Sample and Standard Preparation

Individual standard solutions of gallic acid, tannic acid, catechin, EC, EGC, ECG, EGCG, and procyanidins (B_2_, C_1_, D, and E) were prepared at 1 mg/mL in MilliQ water. Stock solutions were diluted with THF/aqueous LiBr 12 mM (95:5, *v*/*v*) or 0.1% acetic acid water: methanol (60:40, *v*/*v*) solution to use for GPC and MS analysis, respectively. 

For the GPC analysis, Ecovitis™ was dissolved in THF/aqueous LiBr 12 mM (95:5, *v*/*v*) (10 mg/10 mL). Samples were vortexed for 30 s and centrifuged at 10,000× *g* for 10 min, verifying the complete dissolution. The PAC standards were prepared by dilution of stock solution with THF/aqueous LiBr 12 mM (95:5, *v*/*v*). The polystyrene calibration curve, prepared in sets of three vials, each containing four standards spaced across the full molecular weight MW range of the kit, was injected in THF/aqueous LiBr 12 mM (95:5, *v*/*v*).

For LC–MS analysis, 2 mg of Ecovitis™ was weighed in a conic tube, and 2 mL of 0.2% acetic acid in water added. Samples were vortexed until the extract was dissolved entirely. Before the MS analysis, the extract was further diluted with 0.1% acetic acid water: methanol (60:40, *v*/*v*) solution to a final concentration of 20 µg/mL.

### 2.4. GPC Analysis

GPC measurements were performed using an HPLC system (Beckman System Gold 126) with a Beckman 166 UV–VIS detector at 280 nm. A 5 µm, 7.5 mm × 300 mm, 500 Å PLgel Individual Pore Size Column (Agilent PL1110-6525) connected to a precolumn filter 0.5 µm (Upchurch Scientific, WA, USA) was used at room temperature for PACs separation, with an isocratic elution of THF/aqueous LiBr 12 mM (95:5, *v*/*v*) at a flow rate of 1.0 mL/min, according to the method previously described [18,19]. The injection volume was 10 µL, and the acquisition time 11 min.

To test the column response to different molecular weights, the 12-point calibration curve of certified polystyrene (MW range 152–56,600 g/mol) was injected under the same conditions. 

All PAC standard solutions were analyzed at 0.1 mg/mL for the determination of retention times. Epicatechin from 0.025 to 0.125 mg/mL was used for quantitation. 

Samples were injected at 1.0 mg/mL containing BHT 0.3 mg/mL as an internal standard.

### 2.5. LC–MS Analysis

#### 2.5.1. LC–MS Conditions

The flow injection analysis (FIA) mode was adopted on a 1200 series HPLC system through a Chip Cube nano-electrospray ionization ESI interface coupled online with a 6520 quadrupole time-of-flight Q-TOF mass spectrometer (Agilent Technologies, CA, USA) governed by Agilent MassHunter Workstation data acquisition software (B.05.00 version). Sample vials were placed in at 10 °C in the autosampler compartment, and 2 µL were injected onto a FIA-Chip (II) for the analysis. A solution 60:40 (*v*/*v*) of 0.1% acetic acid in Milli-Q water and 0.1% acetic acid in methanol was used as a mobile phase. The flow rate was 0.50 µL/min (from 0 to 3.8 min), at 4.20 min was reduced to 0.35 µL/min (hold for 4.8 min) and brought back to the initial conditions for 3 min (total run time 12 min).

The Q-TOF operated in the negative MS mode at 4 GHz high resolution with three reference mass correction ions to ensure mass accuracy. Data were acquired in the profile full scan mode at a scan rate of 1 spectra/s in the range between 100 and 3200 m/z. The capillary voltage was set to 1600 V (negative polarity) with nitrogen as desolvating gas at 330 °C and 5 L/min; fragmentor, skimmer, and octupole were set at 250, 65, and 750 V, respectively. Acquisition parameters were set at the MS scan rate of 1 spectrum s^−1^ in the range between 130 and 1.700 m/z in the high-resolution mode (*R* = 20.000). 

In data-dependent MS/MS characterization, precursor ions were selected at an isolation width of 4 m/z and fragmented by collision-induced dissociation (CID) at collision energies based on precursor mass and charges. When required, specific targeted precursor masses were selected before analysis and acquired, from 5 to 8 min, at an MS/MS scan rate of 1 spectrum s^−1^ and fragmented at fixed collision energies from 7 to 25 eV with an isolation width of 1.3 m/z.

#### 2.5.2. Procyanidin Identification and Quantification by MS

Data were processed with MassHunter Qualitative Analysis software rel. B07.00 (Agilent Technologies, Santa Clara, CA, USA). The resolved isotope deconvolution method of the Agilent MassHunter data system was used for the charge state deconvolution of the averaged mass spectra. The trial-and-error approach was used to determine the optimal deconvolution parameters. Peak location parameters, maximum spike width was set to 2, and required valley to 0.70. Isotope grouping parameters: peak spacing tolerance was set to 0.00 m/z and 2.0 ppm; the isotope model was set to common organic molecules. The maximum charge state was set to 4. In the spectra, before deconvolution, masses are considered in terms of m/z and after deconvolution in terms of neutral (zero-charge) mass values.

Compounds were extracted from the raw data using the find by molecular feature extraction (MFE) algorithm. The algorithm uses the accuracy of the mass measurements to group related ions related by charge-state envelope, isotopic distribution, and/or the presence of adducts and dimers. It assigns multiple species (ions) that are related to the same neutral molecule (for example, ions representing multiple charge states or adducts of the same neutral molecule) to a single compound that is referred to as a feature. Negatively charged signal from deprotonation and/or acetate adducts, with a charged state up to 3, following the common isotope model were extracted. Masses were extracted from 6 to 7 min, in the range between 160 and 1800 m/z, excluding a signal smaller than 160 counts. The extracted feature list was then queried against the homemade PACs database of monoisotopic mass, and chemical formulas (554 compounds) set to a 50-ppm tolerance.

Four base subunits (catechin, gallocatechin, catechin-3-O-gallate, and gallocatechin-3-O-gallate) were utilized to create the theoretical PAC database of monoisotopic mass and chemical formulas. These four subunits represent the primary grape derived subunits. Theoretical oligomers from all possible combinations of the four units from polymerization of 2 up to 15 were calculated, limiting the maximum number of EGC and galloylated bases to 3 and 5, respectively.

The feature table reported the results of processing the selected sample files, such as ID, sample name, formula, m/z, ppm difference from theoretical mass, number, and charge of ions in the cluster, height, and volume. The compound volume generated by molecular feature extraction, which is formed from the sum of the individual ion abundances within the compound spectrum ions (MFE spectrum) in the specified time window, was used as the parameter for compound abundance. External calibration curves prepared by spiking to matrix EC, B2, C1, D, and E procyanidin standard solutions were used for quantitation.

### 2.6. Statistical Analysis

The graphical and statistical analyses were carried out using OriginPro 2020b (OriginLab, Northampton, MA, USA). Values are means ± std.

## 3. Results

### 3.1. Preparation of the Ecovitis*™*

The GSE Ecovitis^TM^ was produced as described under the Methods from grape pomaces. The outline of the preparation procedure (patent pending) is summarized in Figure 1.

### 3.2. GPC Results

#### 3.2.1. GPC Procedure and Molecular Weight Calibration 

**Figure 2:**
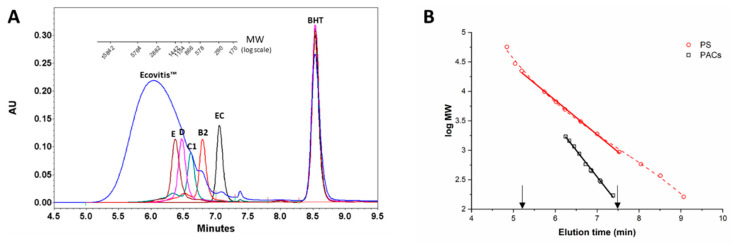
Gel permeation chromatography (GPC) data: (**A**) Gel permeation chromatograms of procyanidin standards (epicatechin EC, dimer B2, trimer C1, tetramer D, and pentamer E) overlaying the chromatogram of Ecovitis™ (blue line). Elution with THF/aqueous LiBr 12 mM (95:5, *v*/*v*). The bar of the corresponding molecular mass over elution time is shown above; (**B**) calibration curves of PACs (GA, TA, EC, ECG, EGC, EGCG, PC-B2, PC-C1, PC-D, PC-E, and black squares) and polystyrene (red circles). For polystyrene, the linear part of the calibration plot (MW range 935–22,290) is shown as solid red line. Arrows indicate the observed linear range for the GPC column.

For the GPC analysis of Ecovitis^TM^ (Figure 2A), the column was calibrated with both polystyrene and PAC individual standards generating the plots of molecular mass distribution (Log scale) over elution time (Figure 2B).

Polystyrene standards in the MW range 56,600–162 Da eluted between 5 and 9 min and were fitted to a polynomial function of 3rd degree. The expected linear region laying between 5.2 and 7.5 min, corresponding to an apparent polystyrene MW range from 22,290 to 935 Da, was used as the operative range to evaluate the molecular weights distribution of PACs. Calibration curve obtained from 10 commercially available standards of polyphenol (GA, TA, EC, ECG, EGC, EGCG, PC-B2, PC-C1, PC-D, and PC-E), spanned a MW range from 1701 to 170 Da, in the elution timeframe from 6.25 to 7.38 min (linear coefficient 0.996). In agreement with a previous report [20], the linearity range determined by polystyrene standards allows the extrapolation of the PAC calibration to the molecular weights of oligomers eluting from 5.2 to 6.25 min.

#### 3.2.2. PACs Quantification by GPC Absorbance Profile at 280 nm 

UV absorbance in GPC analysis can be used to evaluate the extract’s composition when the molar extinction coefficients (ε) of different species are known. The ε of 10 authentic standards (C, EC, EGC, gallic acid, ECG, EGCG, procyanidin B1, procyanidin C1, procyanidin D, and procyanidin E) were calculated from a 5-points calibration curve at 280 nm in the eluent used for GPC. Linear correlation coefficients range was >0.997 passing through zero (Figure S1). As expected, and previously reported [21], ε values of polymers of catechin increase with the degree of polymerization (DP). A polymer of *n* units of catechin has an ε coefficient *n* times higher than catechin (Table 1): it defines the molar absorptivity ratio of a polymer to that of the reference compound (MRRF).

This precise correlation among DP and MRRF in PACs (Table 1) allows the use of GPC profile absorbance at 280 nm to evaluate, in first approximation, the amount at different retention times of PACs at different DP, expressed as micrograms of catechin equivalents (Figure S2).

A limit of this approach is the different ε of catechin gallate (CG/ECG) and gallocatechin (GC/EGC) residues (Table 1). This leads to an overestimation of the catechin equivalents of about 1.8% (in weight) every 1% of catechin gallate (in molarity) and to an underestimation of about 0.75% (in weight) every 1% of gallocatechin (in molarity). Although quantitatively not particularly relevant, this error can be corrected based on the molar fraction of CG/ECG, and GC/EGC calculated from MS data (see Section 3.3.3).

#### 3.2.3. GPC Profile Analysis and Determination of Median Degree of Polymerization (mDP)

**Figure 3:**
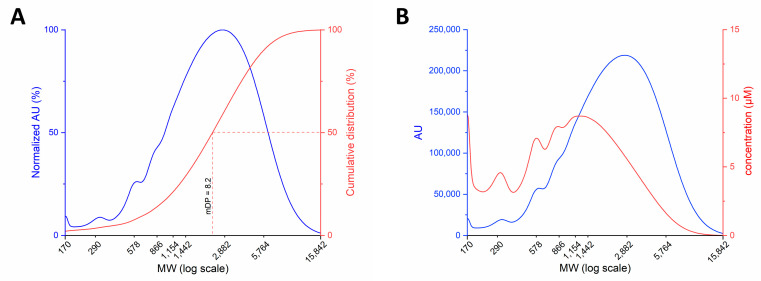
GPC profiles of Ecovitis™ procyanidins as function of their MW: (**A**) blue line: normalized AU (%); red line: cumulative distribution and median degree of polymerization (mDP), expressed as the number of catechin units contained at the average molecular weight; (**B**) blue line: catechin absorbance (AU); red line: concentration (µmolar) of PACs along with the GPC profile. The latter was obtained by dividing the molar concentration of catechin equivalents calculated from absorbance for the DP corresponding to the MW in the plot.

The relationship between the GPC elution profile and PACs molecular weight allows extracting, from the normalized catechin absorbance, the cumulative distribution curve from which the mean degree of polymerization (mDP) of the extract can be calculated (Figure 3A). Mean degree of polymerization is expressed as the ratio between MW corresponding to 50% of the PACs cumulative distribution and the catechin residue’s MW (288 Da) [20]. Ecovitis^TM^ shows a catechin oligomers composition corresponding to a mDP of 8.2. Figure 3B further describes the inferred molar amount of the species in the extract as deduced from the absorbance plot. As expected, the concentration of oligomers sharply decreased at DP > 10.

This gives an account for the difference in sensitivity between GPC and mass spectrometry on the detection of DP > 14 (see Section 3.3.2).

### 3.3. LC–MS Results 

#### 3.3.1. LC–MS Analysis and Calibration

An electrospray ionization high resolution mass spectrometry (ESI-HRMS) procedure was developed based on flow-injection without chromatographic separation. From multiple solvent compositions tested, we selected methanol/water 40/60 solution containing 0.1% acetic acid in respect to both solubilization of sample and stabilization of the multiply charged species without adduct ions formation. The negative-ion mode was selected for higher (>than 3-fold) sensitivity than the positive-ion. Ten species were confirmed with authentic standards; others were identified by exact mass (error < 5 ppm), MS/MS fragmentation pattern and available data from the literature [22]. The deprotonated ion at m/z 169 in the MS/MS spectrum confirmed the identification of gallic acid.

The used shotgun approach cannot resolve the sample’s stereoisomer complexity, descending from catechins’ dual chiral nature, which increases exponentially with the DP [22]. However, missing this information translates in a useful higher detection sensitivity, which is critical for PAC with DP > 7.

**Figure 4:**
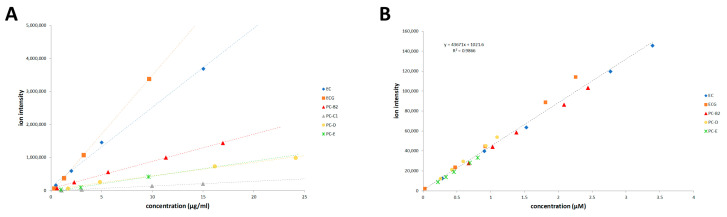
ESI Q-TOF MS matrix-matched calibration curve obtained from linear regression of a single analyte ion volume versus concentration expressed as µg/mL (Plot **A**) and cumulative data from epicatechin (EC), epicatechin gallate (ECG), procyanidin B2, D, and E expressed as µmolar concentration (Plot **B**).

The linear dynamic range of the nanospray ionization was investigated using six standards (EC, ECG, PC-B2, PC-C1, PC-D, and PC-E) in the range from 0.5 to 30 µg/mL. The analysis was carried out both as single analytes and as a mixture of them. The upper concentration limit for the ESI linear dynamic range was approximate to 60, 50, 30, 20, 15, and 8 µM for EC, ECG, B2, C1, D, and E respectively (*R*^2^ > 0.997), corresponding to a maximum of about 20–25 µg/mL of a single analyte (Figure 4A).

From an accurate analysis of the dynamic range of individual species in samples of different complexity, we also observed that the limit of linearity is dictated by the sample’s total catechins content, irrespective of the polymerization degree. When the injected sample concentration was limited to about 25 µg/mL of catechin, the linearity was preserved (Figure S3).

A matrix-matched 5-points calibration curve was made for quantitation by spiking a dissolved grape seed extract solution (10 µg/mL) with standard PACs mixture solution at four different weight equivalent concentrations of 0.2, 0.4, 0.8, and 1 µg/mL. The endogenous concentrations of matrix analytes, calculated from the intercept of the calibration curves, were added to the nominal concentration of spiked standards, to generate the correct concentrations.

The sum of all isotope’s intensity in the compound’s specific isotopic pattern (ion volume) has been used for amount calculations. Calibration curves were plotted using linear regression of the analyte ion volume versus concentration. All the compounds showed correlation coefficients (*R*^2^) higher than 0.999 (Figure S4). The limit of quantitation values ranged from 0.01 to 0.1 μg/mL, depending on DP.

Notably, when all the ions in the isotopic pattern (ion volume) were considered, the six species used for calibration show the same ionization response for mole. Accordingly, a single calibration curve is obtained by plotting the cumulated data from EC, ECG, dimer, tetramer, and pentamer (Figure 4B). This implies that we could assume that all PACs have the same ionization response in our analytical setting, irrespective the DP. Therefore, the regression coefficients obtained from standards were suitable for the quantitative analysis of GSE at higher DP.

#### 3.3.2. MS Profile of Grape Seed Extracts

**Figure 5:**
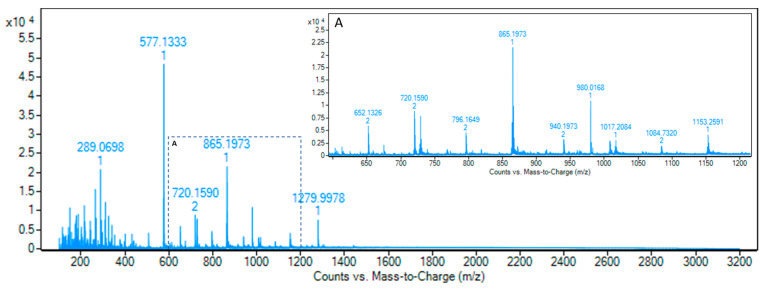
Representative ESI Q-TOF mass spectrum of Ecovitis™. Insert A is the x-axis expanded segment from 600 to 1200 m/z.

A typical ESI mass spectrum of Ecovitis™ is shown in Figure 5. Although the negative spectrum permits the detection of ions between m/z 100 and 3200, no significant ions were detectable above 1700 m/z. The major ions observed at m/z 289, 577, 865, 1153, and 1441 can be attributed to the [M − H]^−^ type-B procyanidin nongalloylated species, with DP between 2 and 5. The presence of minor amounts of ions with two mass units less than the corresponding ion is tentatively attributed to a [M − H]^−^ type-A procyanidin. The ions of type-B procyanidin mono-galloylated species were identifiable at m/z 441, 729, 1017. Moreover, the zoomed m/z area (A) in Figure 5 shows the presence of multiple, mainly double and triple, charged deprotonated molecules ([M − 2H]^2−^ and [M − 3H]^3−^). 

**Figure 6:**
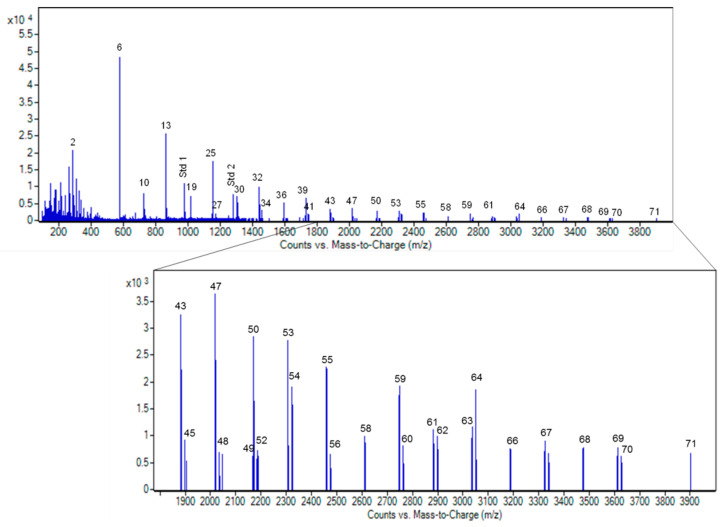
Deconvoluted ESI Q-TOF mass spectrum of Ecovitis™ grape seed extract. Compounds are identified with ID numbers as in Table 2.

Multiple charged ions are most frequently produced from oligomers with DP higher than 3 [23]. Although the presence of multiple charges states increased the sensitivity, this, together with the partial isotope pattern overlapping due to the presence of both A- and B-type species, made more complex the interpretation of the spectrum. This can be overridden using the deconvolution algorithm that transforms a charge state series into a singular molecular mass. The deconvoluted spectrum of Ecovitis™ grape seed extract is reported in Figure 6. Notably, oligomers up to DP 13 were detected, matching the highest range so far reported for MS analysis of PACs [23].

#### 3.3.3. Quantitative Analysis of Ecovitis™ Components

PACs analysis was carried out in triplicate, by flow injection of 2 µL of a solution of 20 µg/mL of Ecovitis™. Signals are deconvoluted and matched vs. a homemade PACs database generated as described in Section 2.5.2. Each identified compound was quantified by its ion volume vs. the multi-PAC calibration curve (Figure 4) described in Section 3.3.1. A total of 71 compounds were identified and quantified until approximately 1 µg/mg of dry extract (Table 2). When the shotgun approach does not precisely separate isomers, the compounds are cumulatively indicated. For the same reason, the possible regio-isomers formed by the gallate associated to a PAC with DP ≥ 2 cannot be deduced. Additionally, there was a close match between 1 gallocatechin and 2 gallate residues that differed by only 0.035 mDa. Although this difference was enough to be discriminated by our instrument, we consistently found an error higher than average (>6 ppm), strongly suggesting both species’ copresence. Since the MS/MS fragmentation pattern confirmed the occurrence of both compounds, these were independently reported in the table.

#### 3.3.4. Integration of Information from GPC and MS

As previously pointed out, the definition by UV detection of the pattern of the composition of polymers of PACs preparation (Figure 2) was biased by the different molar extinction coefficients of catechin, catechin-gallate, and gallocatechin. Although quantitatively not particularly relevant in GSE, this bias can be corrected using data from MS.

From the extended coverage of PACs quantification by MS, we could deduce the molar fraction of the three primary residues producing different PAC by polymerization. The molar fraction of catechin, catechin-gallate, and gallocatechin polymers in Ecovitis™ resulted in 0.88, 0.11, and 0.015, respectively. This permitted the correction of the PACs content produced by UV absorbance with the information made by MS from 931.3 to 792.7 µg/mg of powder.

As expected, the total PACs content obtained from MS was lower due to the limit of detectability at approximately 3600 MW. The whole content calculated in Ecovitis™ from data in Table 2 resulted in 598.3 µg/mg. This indicated that about 20% of PACs at the highest MW and lowest molar concentration escaped the MS identification.

Nonetheless, the overall procedure was validated by the evidence of a good correspondence between PACs content by GPC and MS compared to the range of detectability by MS. In the span of MW 160–1450, including all original standards, the values were 261.1 and 284.4 µg/mg for GPC and MS analysis, respectively. In the range 160–3600, the values were 579.4 and 592.6 µg/mg, respectively.

This cross-validated the two approaches and permitted the accurate, although approximated, definition of species’ quantity at different MW from the cumulative distribution reported in Figure 3A.

#### 3.3.5. Application of the Integrated Analytical Procedure to a Benchmark of GSE

The complete analytical procedure including GPC and UV detection and MS was tested on a typical benchmark of GSE. We used Enovita^®^ manufactured by Indena S.A.S.

While the two GSE were indistinguishable by FTIR (Figure S6), Enovita^®^ differentiates from Ecovitis™ in respect to the higher content of lower MW components, including the monomers and the lower content of species at high DP (Figure S7 and Table S1).

## 4. Discussion

The grape seed extract (GSE) was almost exclusively composed of proanthocyanidins, known as catechol-type non-hydrolyzable tannins. These are flavan 3-ol derivatives polymers and galloylated esters. Analytic characterization of GSE encompasses colorimetric assays, TLC, HPLC, NMR, and MS approaches, none of which, alone, reaches the goal of the comprehensive definition of all its features. On the other hand, this information is relevant regarding the identification and definition of quality of a given GSE and prevention of adulteration [13].

An innovative technology was introduced to produce the GSE Ecovitis^TM^. In the industrial process, a supply chain of fresh pomaces from wineries in the North East of Italy was used. The adopted tangential flow filtration technology uses only water as the solvent and generates the GSE as a dry powder that fits the usual routine test of typical GSE.

However, the preparation process’s novelty motivated the definition of more in-depth characterization of GSE to reach both rapid but accurate descriptions of the product, suitable for quality control procedures and precise definition of Ecovitis^TM^ in respect to other GSEs available on the market. In this respect, we used as a reference and suitable benchmark Enovita^®^ manufactured from Indena S.A.S.

We reached an accurate description of features by integrating and cross-validating results obtained from GPC and flow injection ESI Q-TOF MS technology.

In GPC, PACs species at DP > 3 largely overlap. We first deduced, adopting an accurate calibration with available original standards, a linear fitting of all species’ retention time possibly present in GSE. We analytically confirmed that ε of different polymers corresponded to that of catechin times the polymerization level (*n*). From these two sets of information, we processed the absorbance vs. time graph of GPC in two plots allowing the definition of two relevant and discriminant features of a GSE: (i) the distribution and concentration of species at different MW and (ii) the mean value of polymerization.

The intrinsic bias of the approach is the different ε of galloylated catechins and gallic esters. This bias impacting for approximately 15% in evaluating PACs’ total content in the extract can be corrected, knowing the molar fraction of catechins, gallocatechin, and catechin gallates deduced from quantitative MS analysis.

The MS fingerprint of PACs composition of Ecovitis^TM^ was obtained by a procedure based on flow-injection without chromatographic separation. A drawback of the adopted shotgun approach is the missed possibility of resolving the sample’s stereoisomer complexity, which increases with the DP. On the other hand, this approach largely increased the sensitivity at high polymerization degrees and the signal over concentration linearity. The latter also contributed to the adopted use of the sum of isotope’s intensity in the specific isotopic pattern (ion volume). Optimal linearity between the concentration of a compound and signal was experimental validated for standards up to *n* = 5 and extrapolated to the whole GSE provided a threshold is not exceeded of total catechins content at different polymerization levels. This approach permitted the quantification of more than 70 species up to *n* = 13.

MS data also produced the molar fraction values used to correct GPC data for the presence of gallocatechin and catechin gallates.

In summary, this integrated analytical procedure was set up to characterize the features of the new GSE Ecovitis^TM^ produced by the innovative filtration procedure. Still, it was also proposed as a suitable and straightforward integrated approach to describe different PACs. 

Data from GPC describe polymers’ presence up to an n value of 30, while the detection by MS was restricted to *n* = 13 due to the low concentration of oligomers with DP > 14 (see Figure 3B). Moreover, the increase of DP is associated with a parallel expansion in the percentage of galloylated oligomers and the isotopic pattern complexity [22,23] further contributes to limit the possibility.

Although this is an intrinsic limit of the integrated approach, we must consider that, for the typical detection range (or detected species), data obtained by GPC were in full agreement with data produced by MS. However, MS did not confirm approximately 20% of species at the highest DP. 

The analytical procedure described was used for monitoring the production of Ecovitis^TM^ and quality control. The distribution of polymers at different molecular weight in four large scale preparation (each up to 150 kg) produced over four months indicates only a minimal variation in monomers’ relative content (Figure S5).

From the comparison with the GSE benchmark Enovita^®^, Ecovitis^TM^ indicates that, despite the same FTIR spectrum (Figure S6), the latter had a higher median degree of polymerization (Figure S7 and Table S1).

## 5. Conclusions

The GSE Ecovitis^TM^ was produced by an innovative technology and characterized by an integrated analytical approach of GPC and MS. The emerging feature of Ecovitis™ vs. an established benchmark in the market, produced by a different technology, is the much lower content of species at low DP and the corresponding increase of species at high DP. Future studies are needed to decipher the actual differences of having longer polymers and low monomer content regarding the known health benefits of GSE.

## 6. Patents

Processo di preparazione di estratti da vinacciolo ed estratti così ottenuti (Filing date: 31 December 2020).

## Figures and Tables

**Table 1:** Retention times and molar extinction coefficient of PAC standards analyzed by GPC.

Standard	MW (g/mol)	DP ^1^	λmax (nm)	ε 280 nm ^2^ (M^−1^ cm^−1^)	MRRF ^3^	GPC Rt ^4^ (min)
(+)-Catechin	290.271	1	279	3950	1.00	7.09
(−)-Epicatechin	290.271	1	279	3950	1.00	7.09
Procyanidin B2	578.526	2	279	8050	2.00	6.78
Procyanidin C1	866.778	3	279	12,130	3.00	6.62
Procyanidin D	1155.02	4	279	16,075	4.00	6.48
Procyanidin E	1443.27	5	279	20,470	5.00	6.38
EGC/GC	306.27	1	279	1200	0.31	7.07
ECG/CG	442.376	2	278	13,500	3.40	6.87
EGCG/GCG	458.375	2	274	10,600	2.30	6.85
Gallic acid	170.120	1	273	8500	2.15	7.38

^1^ DP: degree of polymerization; ^2^ **ε**: extinction coefficient, calculated with calibration at 280 nm (*R*^2^ = 0.999); ^3^ MRRF: molar absorptivity ratio of the compound to that of a reference compound; ^4^ Rt: GPC retention time at 280 nm. EGC: epigallocatechin; GC: gallocatechin; ECG: epicatechin-gallate; CG: catechin-gallate; EGCG: epigallocatechin-gallate; GCG: gallocatechin-gallate.

**Table 2:** Proanthocyanidins identified in Ecovitis™ by LC-Chip/ESI-Q-TOF–MS analysis performed in the negative ion mode. The concentration of compounds, as µg/mg of extract, are the average obtained from triplicate analyses.

Ecovitis™ Proanthocyanidins											
ID	Compound	MW	Mean	SD	ppm	ID	Compound	MW	Mean	SD	ppm
**1**	G	170.0215	ND			**37**	PA (A) 4EC 1EGC 1G	1608.3228	1.79		1.9
**2**	C/EC	290.079	9.51	0.23	−4.3	**38**	PA (B) 4EC 1EGC 1G	1610.3385	2.59	0.67	−3.0
**3**	EGC	306.0739	ND			**39**	PC (B) 6EC	1730.3960	25.12	0.66	1.4
**4**	ECG	442.0900	2.76	0.05	2.9	**40**	PA (A) 5EC 1EGC	1744.3752	2.84	0.10	−0.4
**5**	EGCG	458.0849	ND			**41**	PC (B) 5EC 2G	1746.3545	10.62	0.20	3.8
**6**	PC (B) 2EC	578.1424	49.85	1.89	−0.7	**42**	PC (A) 6EC 1G	1880.3913	2.43	0.06	0.5
**7**	PA (A) 1EC 1EGC	592.1217	1.06	0.06	8.4	**43**	PC (B) 6EC 1G	1882.4070	23.57	0.59	1.8
**8**	PA (B) 1EC 1EGC	594.1373	1.20		−0.7	**44**	PA (A) 5EC 1EGC 1G	1896.3862	1.24	1.76	1.6
**9**	ECG-Glu	604.1428	1.62	0.09	−0.8	**45**	PA (B) 5EC 1EGC 1G	1898.4019	4.29	0.17	−2.7
**10**	PC (B) 2EC 1G	730.1534	11.44	0.36	0.3	**46**	PC (A) 7EC	2016.4437	2.92	0.08	−1.2
**11**	PA (A) 1EC 1EGC 1G	744.1326	ND			**47**	PC (B) 7EC	2018.4594	23.83	0.82	1.9
**12**	PA (B) 1EC 1EGC 1G	746.1483	ND			**48**	PC (B) 6EC 2G	2034.4179	7.65	0.32	3.5
**13**	PC (B) 3EC	866.2058	46.36	1.77	0.7	**49**	PC (A) 7EC 1G	2168.4547	2.31	0.18	0.5
**14**	PA (A) 2EC 1EGC	880.1851	1.65		7.4	**50**	PC (B) 7EC 1G	2170.4703	17.51	2.72	1.9
**15**	PA (B) 2EC 1EGC	882.201/882.164	1.89	0.03	−17.3/22.7	**51**	PA (A) 6EC 1EGC 1G	2184.4496	ND		
**16**	PA (A) 1EC 2EGC	896.1800	0.90	0.03	12.9	**52**	PA (B) 6EC 1EGC 1G	2186.4653	4.12	0.15	−2.5
**17**	PA (B) 1EC 2EGC	898.1956	ND			**53**	PC (B) 8EC	2306.5228	11.74	4.53	0.8
**18**	PC (A) 3EC 1G	1016.2011	ND			**54**	PC (B) 7EC 2G	2322.4813	13.87	0.38	2.3
**19**	PC (B) 3EC 1G	1018.2168	16.35	0.41	0.6	**55**	PC (B) 8EC 1G	2458.5337	8.77	0.29	1.9
**20**	PA (A) 2EC 1EGC 1G	1032.1960	1.00	0.05	17.3	**56**	PA (B) 7EC 1EGC 1G	2474.5286	1.74	0.10	−8.8
**21**	PA (B) 2EC 1EGC 1G	1034.2117	ND			**57**	PC (B) 9EC	2594.5862	8.66	0.25	0.3
**22**	PA (A) 1EC 2EGC 1G	1048.1910	ND			**58**	PA (B) 8EC 1EGC	2610.5811	11.13	0.32	−1.6
**23**	PA (B) 1EC 2EGC 1G	1050.2066	ND			**59**	PC (B) 9EC 1G	2746.5970	10.90	0.15	−0.3
**24**	PC (A) 4EC	1152.2536	4.50	0.23	−0.8	**60**	PA (B) 8EC 3G	2762.5556	5.00	0.21	3.95
**25**	PC (B) 4EC	1154.2692	42.47	2.24	0.2	**61**	PC (B) 10EC	2882.6496	9.89	0.14	0.3
**26**	PA (A) 3EC 1EGC	1168.2485	3.25	0.09	1.3	**62**	PC (B) 9EC 2G	2898.6081	7.68	0.18	1.3
**27**	PA (B) 3EC 2G	1170.2277	5.44	0.29	6.9	**63**	PC (B) 10EC 1G	3034.6605	6.05	0.16	2.7
**28**	PA (B) 2EC 1EGC 2G	1186.2227	ND			**64**	PA (B) 9EC 3G	3050.6190	9.89	0.24	0.62
**29**	PC (A) 4EC 1G	1304.2645	1.92	0.42	0.9	**65**	PC (B) 11EC	3170.7129	7.99	0.31	1.3
**30**	PC (B) 4EC 1G	1306.2802	25.12	0.69	1.2	**66**	PC (B) 10EC 2G	3186.6715	6.72	0.01	1.5
**31**	PC (A) 5EC	1440.3169	3.41	0.13	−1.1	**67**	PC (B) 11EC 1G	3322.7239	10.81	1.00	1.6
**32**	PC (B) 5EC	1442.3326	42.74	1.20	1.1	**68**	PA (B) 11EC 1EGC	3474.7712	6.97	1.51	−1.7
**33**	PA (A) 4EC 1EGC	1456.3119	3.31	0.16	−0.5	**69**	PC (B) 12EC 1G	3610.7873	6.52	1.02	0.3
**34**	PC (B) 4EC 2G	1458.2911	6.66	0.10	4.0	**70**	PA (B) 11EC 3G	3626.7458	4.50	0.47	0.2
**35**	PC (A) 5EC 1G	1592.3279	2.32	0.10	0.4	**71**	PC (B) 13EC 1G	3898.8507	5.74	1.02	0.3
**36**	PC (B) 5EC 1G	1594.3436	24.20	0.11	1.3						

## Data Availability

The data presented in this study are available on request from the corresponding author.

## Supplementary Materials

The following are available online at https://www.mdpi.com/2076-3921/10/3/418/s1, Figure S1: UV calibration curves of PACs; Figure S2: Calibration curve of (−) epicatechin in the GPC-UV system; Figure S3: Effect of sample complexity to the dynamic range of PACs in ESI-MS-Q-TOF; Figure S4: ESI-Q-TOF matrix-matched calibration curve; Figure S5: Concentration of PACs along GPC profile in 4 independent preparations of Ecovitis™; Figure S6: FTIR spectra of Ecovitis™ and Enovita^®^; Figure S7: Cumulative distribution and median degree of polymerization (mDP) of Ecovitis™ and Enovita^®^; Table S1: Proanthocyanidins identified in Ecovitis™ and Enovita^®^.
