# Supplementary material for: Grape Seeds Proanthocyanidins: Advanced Technological Preparation and Analytical Characterization
Source: Antioxidants (Basel). 2021 Mar 9;10(3):418. doi: 10.3390/antiox10030418 (PMC8001487; doi:10.3390/antiox10030418)
Supplement: Supplementary file 1 [file antioxidants-10-00418-s001.pdf]

antioxidants

MDPI

SUPPLEMENTARY DATA

Grape seeds proanthocyanidins: advanced technological preparation and analytical characterization

Paolo Morazzoni [1], Paola Vanzani [2] Sandro Santinello[1], Antonina Gucciardi[2,3], Lucio Zennaro[2], Giovanni Miotto[2,3,4]* and Fulvio Ursini[2]*

1 Distillerie Bonollo Umberto S.p.A., Nutraceutical Division, Mestrino, Padova, Italy
2 Department of Molecular Medicine, University of Padova, Italy
3 Proteomics Center, University of Padova and Azienda Ospedaliera di Padova, Padova, Italy
4 CRIBI Biotechnology Center, University of Padova, Padova, Italy
* Corresponding Authors: FU, fulvio.ursini@unipd.it; GM, giovanni.miotto@unipd.it

Supplementary Figures

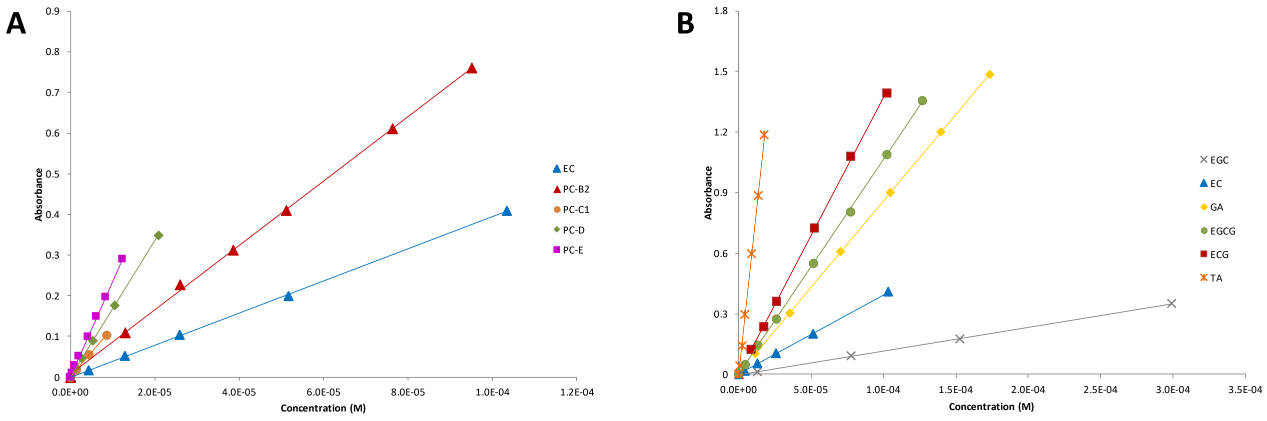

Figure 1. UV calibration curves of PACs.

Calibration curve of PACs standards at 280 nm in GPC medium (THF/aqueous LiBr 12mM 95:5, v/v) were used to calculate the molar extinction coefficients (ε). The 10 standards are plotted in two separate graphs for clarity, both reporting epicatechin (EC) for comparison. **Plot A**: epicatechin (EC), procyanidin B1, procyanidin C1, procyanidin D, procyanidin E; **Plot B**: gallic acid (GA), epicatechin gallate (ECG), epigallocatechin gallate (EGCG), epigallocatechin (EGC), tannic acid (TA) and EC.

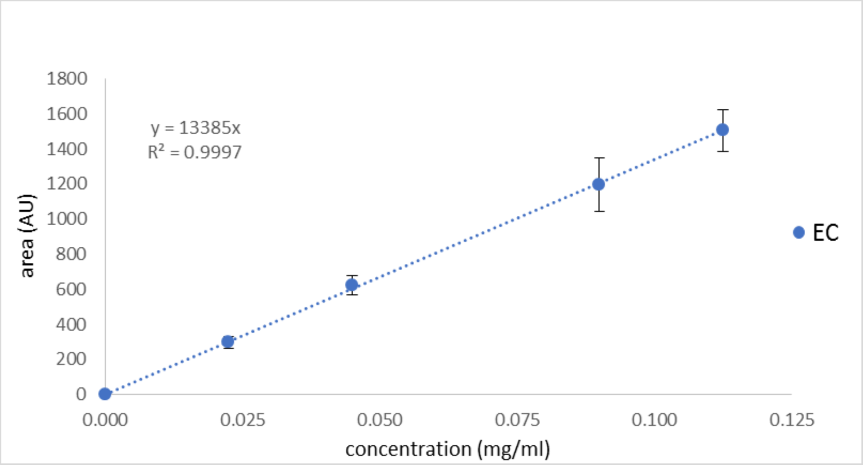

Figure S2. The calibration curve of standard (-)-epicatechin (EC) in GPC-UV system.

The amounts of PACs measured in GPC were obtained from epicatechin calibration straight line (R^2 =0.999) from 0.025 to 0.125mg/mL in THF/aqueous LiBr 12mM (95:5, v/v). All PACs were considered as catechin since their molar extinction coefficient ε (Table 1) was proportional to the number of polymerized units.

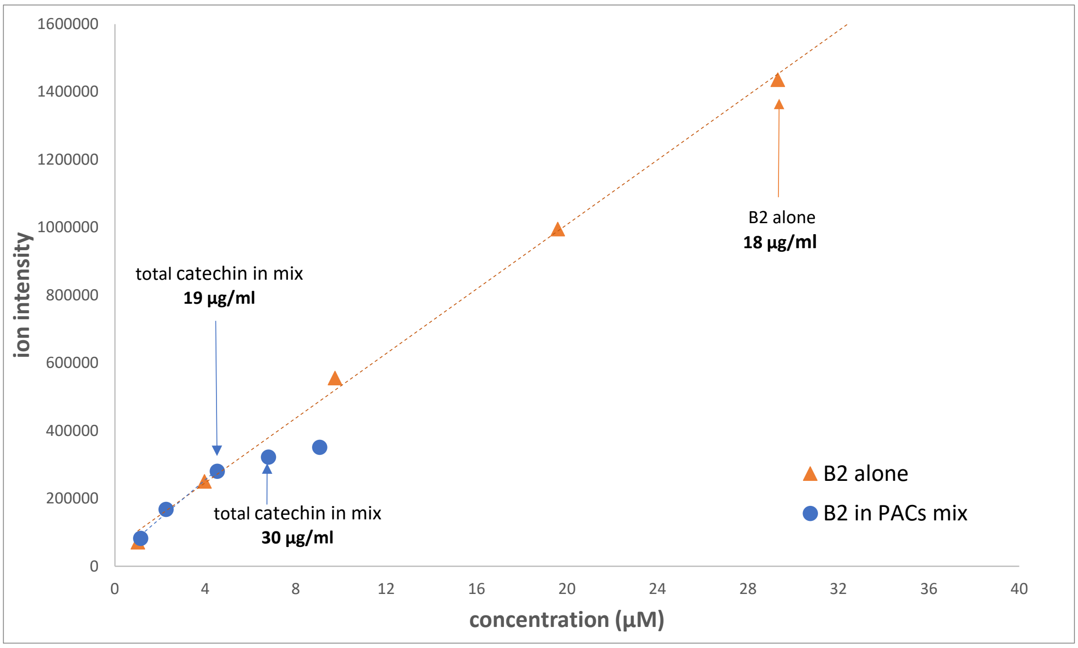

Figure S3. Effect of sample complexity to the dynamic range of PACs in ESI-MS-QTof.

Ion signal intensity of PC-B2 alone solution (orange triangles) versus PC-B2 in a PACs mixture composed of equimolar concentrations of EC, ECG, PC-B2, PC-C1, PC-D, and PC-E (blue circles).

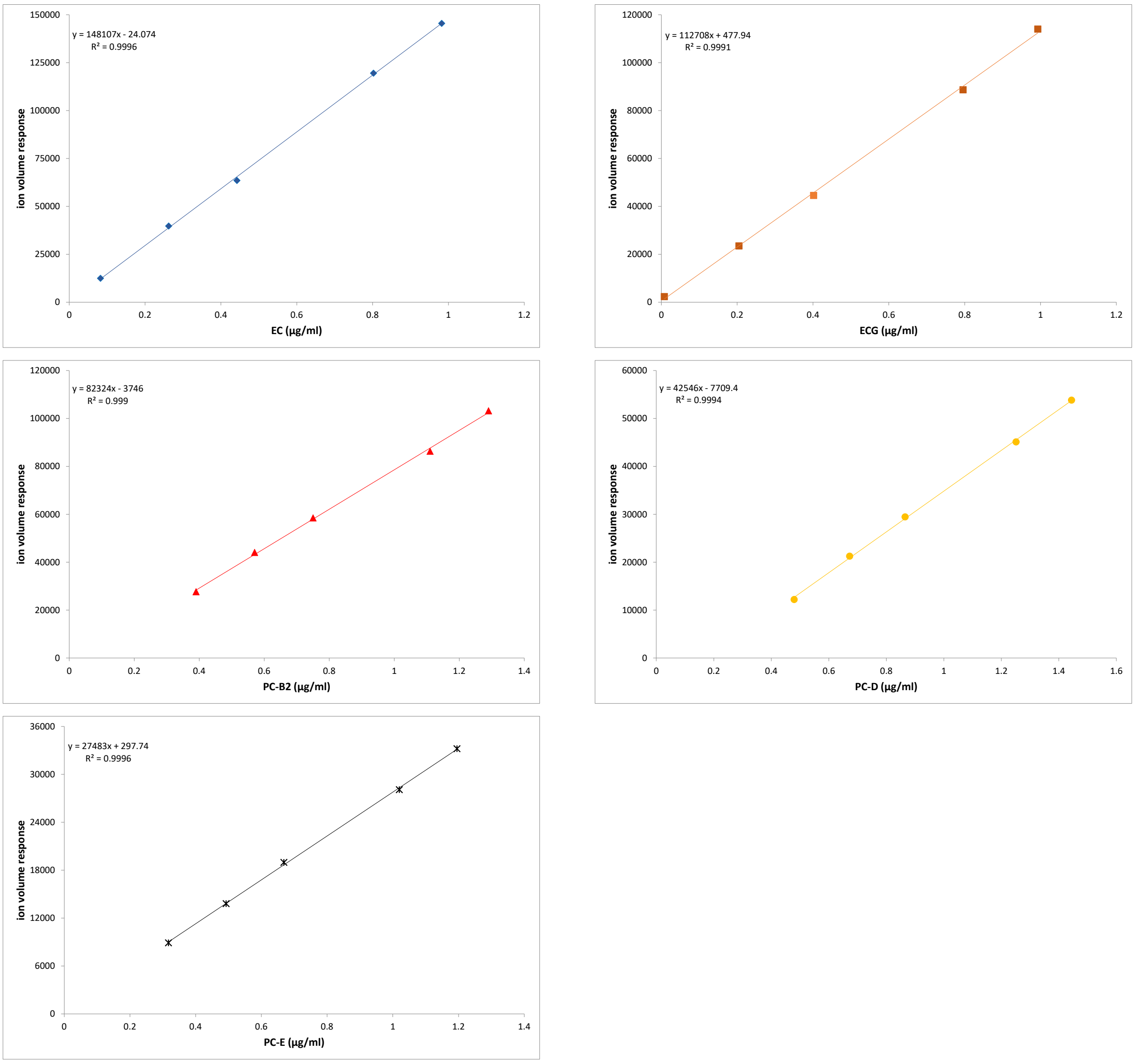

Figure S4. ESI-QTof matrix-matched calibration curve.

Calibration curve were made by spiking a dissolved grape seed extract solution (10μg/ml) with standard PACs mixture solution at four different weight equivalent concentrations (0.2, 0.4, 0.8, 1 μg/mL). The endogenous concentrations of matrix analytes were added to the nominal concentration of spiked standards to generate the correct concentrations.

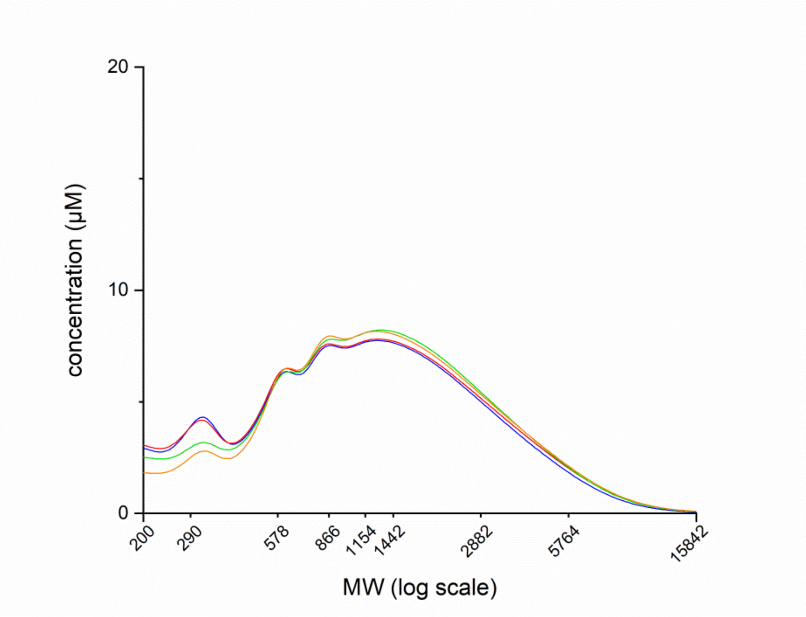

Figure 5. Concentrations of PACs along GPC profile of four independent preparations of Ecovitis™. Micromolar concentrations measured in GSEs industrially produced over four months. Concentration was obtained by dividing the molar concentration of catechin equivalents calculated from absorbance for the DP corresponding to the MW in the plot.

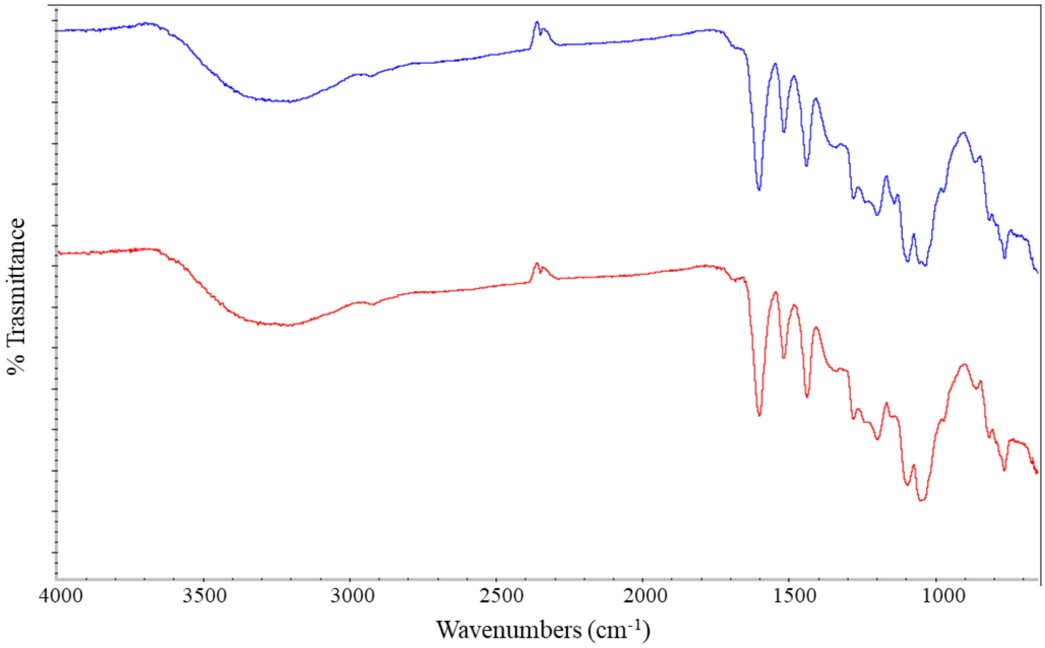

Figure S6. FTIR spectra of Ecovitis™ (red line) versus Enovita® (blue line).

ATR-FTIR measurements were performed using Nicolet Nexus FT-IR of Thermo Nicolet Electron Corporation with OMNIC 32 software. The dried grape seeds powders were pressed on top of the ZnSe crystal. A 64-scan was collected with a 4 cm^{-1} resolution in the range of 4000–650 cm^{-1} at room temperature with automatic atmosphere suppression.

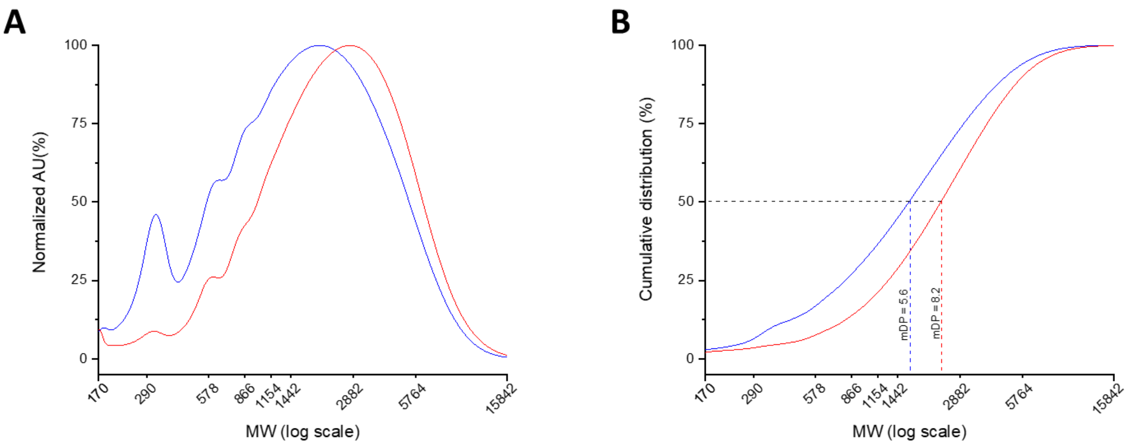

Figure S7. Cumulative distribution and median degree of polymerization (mDP) of Ecovitis™ (red line, mDP 8.2) and Enovita®™ (blue line, mDP 5.6) grape seed extracts.

Supplementary Table

Table S1. Proanthocyanidins identified in Ecovitis™ and Enovita® grape seeds extracts by LC-Chip/ESI-QTOF-MS analysis performed in negative ion mode. Concentrations of compounds, as μg/mg of extract, are average obtained from triplicate analyses.

ID	Compound	MW	Enovita®			Ecovitis™		
			mean	stdev	*ppm*	mean	stdev	*ppm*
1	G	170.0215	1.42	0.05	*-11.2*			
2	C/EC	290.079	87.04	3.00	*-6.0*	9.51	0.23	*-4.3*
3	EGC	306.0739	2.06	0.39	*-7.7*			
4	ECG	442.0900	5.50	1.13	*-3.0*	2.76	0.05	*2.9*
5	EGCG	458.0849	1.54	0.77	*6.8*			
6	PC (B) 2EC	578.1424	70.65	3.08	*-3.6*	49.85	1.89	*-0.7*
7	PA (A) 1EC 1EGC	592.1217	5.36	0.18	*2.6*	1.06	0.06	*8.4*
8	PA (B) 1EC 1EGC	594.1373	3.23	0.18	*-5.4*	1.20		*-0.7*
9	ECG-Glu	604.1428	3.14	0.16	*-7.5*	1.62	0.09	*-0.8*
10	PC (B) 2EC 1G	730.1534	21.56	0.92	*-2.6*	11.44	0.36	*0.3*
11	PA (A) 1EC 1EGC 1G	744.1326	2.05	0.05	*13.0*			
12	PA (B) 1EC 1EGC 1G	746.1483	1.43	0.05	*1.9*			
13	PC (B) 3EC	866.2058	48.02	1.89	*-2.1*	46.36	1.77	*0.7*
14	PA (A) 2EC 1EGC	880.1851				1.65		*7.4*
15	PA (B) 2EC 1EGC/2EC 2G	882.201/ 882.164	3.28	0.01	*-16.9/22.7*	1.89	0.03	*-17.3/22.3*
16	PA (A) 1EC 2EGC	896.1800				0.90	0.03	*12.9*
17	PA (B) 1EC 2EGC	898.1956	1.29	0.04	*13.6*			
18	PC (A) 3EC 1G	1016.2011	0.9	0.30	*8.5*			
19	PC (B) 3EC 1G	1018.2168	20.09	0.91	*-0.7*	16.35	0.41	*0.6*
20	PA (A) 2EC 1EGC 1G	1032.1960	3.21	0.03	*-0.8*	1.00	0.05	*17.3*
21	PA (B) 2EC 1EGC 1G	1034.2117	1.39	0.05	*-1.3*			
22	PA (A) 1EC 2EGC 1G	1048.1910	1.03	0.03	*19.6*			
23	PA (B) 1EC 2EGC 1G	1050.2066	1.04	0.04	*5.8*			
24	PC (A) 4EC	1152.2536	6.01	0.33	*-2.0*	4.50	0.23	*-0.8*
25	PC (B) 4EC	1154.2692	30.00	2.00	*-1.7*	42.47	2.24	*0.2*
26	PA (A) 3EC 1EGC	1168.2485	5.68	0.29	*-0.9*	3.25	0.09	*1.3*
27	PA (B) 3EC 2G	1170.2277	7.81	0.06	*7.1*	5.44	0.29	*6.9*
28	PA (B) 2EC 1EGC 2G	1186.2227	1.15	0.02	*12.5*			
29	PC (A) 4EC 1G	1304.2645	3.79	0.14	*1.4*	1.92	0.42	*0.9*
30	PC (B) 4EC 1G	1306.2802	22.25	0.71	*-0.7*	25.12	0.69	*1.2*
31	PC (A) 5EC	1440.3169	3.35	0.14	*-2.1*	3.41	0.13	*-1.1*
32	PC (B) 5EC	1442.3326	26.76	1.39	*-0.6*	42.74	1.20	*1.1*
33	PA (A) 4EC 1EGC	1456.3119	5.51	0.17	*-4.5*	3.31	0.16	*-0.5*
34	PC (B) 4EC 2G	1458.2911	8.23	0.77	*2.7*	6.66	0.10	*4.0*

35	PC (A) 5EC 1G	1592.3279	2.84	0.13	*-1.1*	2.32	0.10	*0.4*
36	PC (B) 5EC 1G	1594.3436	18.29	0.56	*-0.4*	24.20	0.11	*1.3*
37	PA (A) 4EC 1EGC 1G	1608.3228	2.95	0.08	*1.3*	1.79		*1.9*
38	PA (B) 4EC 1EGC 1G	1610.3385	3.66	0.69	*-4.4*	2.59	0.67	*-3.0*
39	PC (B) 6EC	1730.3960	13.09	0.41	*-1.1*	25.12	0.66	*1.4*
40	PA (A) 5EC 1EGC	1744.3752	3.70	0.17	*-2.5*	2.84	0.10	*-0.4*
41	PC (B) 5EC 2G	1746.3545	11.20	0.49	*3.0*	10.62	0.20	*3.8*
42	PC (A) 6EC 1G	1880.3913	2.80	0.09	*-1.1*	2.43	0.06	*0.5*
43	PC (B) 6EC 1G	1882.4070	15.40	0.30	*-0.3*	23.57	0.59	*1.8*
44	PA (A) 5EC 1EGC 1G	1896.3862	3.24	0.09	*1.0*	1.24	1.76	*1.6*
45	PA (B) 5EC 1EGC 1G	1898.4019	5.86	0.19	*-2.9*	4.29	0.17	*-2.7*
46	PC (A) 7EC	2016.4437	3.36	0.80	*-3.2*	2.92	0.08	*-1.2*
47	PC (B) 7EC	2018.4594	6.93	0.21	*-0.5*	23.83	0.82	*1.9*
48	PC (B) 6EC 2G	2034.4179	6.83	0.32	*1.9*	7.65	0.32	*3.5*
49	PC (A) 7EC 1G	2168.4547	2.44	0.07	*0.7*	2.31	0.18	*0.5*
50	PC (B) 7EC 1G	2170.4703	10.57	0.21	*-0.6*	17.51	2.72	*1.9*
51	PA (A) 6EC 1EGC 1G	2184.4496	2.55	0.09	*1.5*			
52	PA (B) 6EC 1EGC 1G	2186.4653	4.98	0.17	*-4.3*	4.12	0.15	*-2.5*
53	PC (B) 8EC	2306.5228	4.24	0.24	*-1.4*	11.74	4.53	*0.8*
54	PC (B) 7EC 2G	2322.4813	5.42	0.80	*1.7*	13.87	0.38	*2.3*
55	PC (B) 8EC 1G	2458.5337	5.89	0.74	*0.1*	8.77	0.29	*1.9*
56	PA (B) 7EC 1EGC 1G	2474.5286	2.45	0.26	*-6.8*	1.74	0.10	*-8.8*
57	PC (B) 9EC	2594.5862				8.66	0.25	*0.3*
58	PA (B) 8EC 1EGC	2610.5811	6.57	0.12	*-0.1*	11.13	0.32	*-1.6*
59	PC (B) 9EC 1G	2746.5970	7.01	0.11	*0.6*	10.90	0.15	*-0.3*
60	PA (B) 8EC 3G	2762.5556	6.85	0.17	*4.6*	5.00	0.21	*3.95*
61	PC (B) 10EC	2882.6496	6.55	0.21	*-11.9*	9.89	0.14	*0.3*
62	PC (B) 9EC 2G	2898.6081	6.77	0.16	*1.9*	7.68	0.18	*1.3*
63	PC (B) 10EC 1G	3034.6605	3.25	0.10	*-3.0*	6.05	0.16	*2.7*
64	PA (B) 9EC 3G	3050.6190				9.89	0.24	*0.62*
65	PC (B) 11EC	3170.7129				7.99	0.31	*1.3*
66	PC (B) 10EC 2G	3186.6715	6.15	0.12	*0.9*	6.72	0.01	*1.5*
67	PC (B) 11EC 1G	3322.7239				10.81	1.00	*1.6*
68	PA (B) 11EC 1EGC	3474.7712				6.97	1.51	*-1.7*
69	PC (B) 12EC 1G	3610.7873				6.52	1.02	*0.3*
70	PA (B) 11EC 3G	3626.7458				4.50	0.47	*0.2*
71	PC (B) 13EC 1G	3898.8507				5.74	1.02	*0.3*
